# Picosecond Laser Direct Writing of Micro-Nano Structures on Flexible Thin Film for X-Band Transmittance Function

**DOI:** 10.3390/ma18020403

**Published:** 2025-01-16

**Authors:** Jiecai Feng, Jin Zhou, Cuilian Xu, Bingdong Yang, Ze Tian, Hongfei Liu, Yilian Zhang, Zhenghao Sun, Xiaohai Peng, Yingzhong Tian

**Affiliations:** 1 School of Mechatronic Engineering and Automation, Shanghai University, Shanghai 200444, China; 2 Department of Basic Sciences, Air Force Engineering University, Xi’an 710051, China; 3 AVIC Manufacturing Technology Institute, Beijing 100024, China; 4 Laser Micro/Nano Fabrication Laboratory, School of Mechanical Engineering, Beijing Institute of Technology, Beijing 100081, China; 5 Shanghai Institute of Spacecraft Equipment, Shanghai 200240, China

**Keywords:** flexible thin film, picosecond laser, laser direct writing, microstructures, X-band transmittance

## Abstract

Recently, ultrafast laser direct writing has become an effective method for preparing flexible films with micro-nano structures. However, effective control of laser parameters to obtain acceptable micro-nano structures and the effect of micro-nano structure sizes on function of the film remain challenges. Additionally, flexible films with high X-band transmittance are urgently required in aerospace and other fields. In this work, we evaluate the feasibility of applying picosecond laser direct writing for fabricating micro-nano structures on the surface of flexible thin film and the relationship between the size of square columnar micro-nano structures and the transmittance of the flexible thin film. The results show that an array of square columnar micro-nano structures was achieved by picosecond laser direct writing on the surface of flexible thin film (Au-SiO_2_-PI) with a thickness of 50 µm. Additionally, excellent micro-nano structures morphology of the square columnar arrays without burning through or destroying were obtained by laser direct writing with a pulse power and frequency of 2 W and 100 KHz, respectively. The results also show that the X-band transmittance was effected by the characteristic of the square columnar array on the surface of the flexible thin film. The X-band transmittance was significantly increased by decreasing the length of the square column on the surface of the flexible thin film. The X-band transmittance was slightly increased by decreasing the width of the groove of the square column on the surface of the flexible thin film.

## 1. Introduction

The unique microscopic morphology of the surface micro-nano structures enables the original material to acquire new physical and chemical properties [1]. The improvement in its optical [2], mechanical [3], electrical [4], magnetic [5], and other properties can enrich the function of the material, so that the original material can obtain a wider application prospect. Therefore, the method for preparing surface micro-nano structures with excellent properties has become the focus of researchers in recent years.

In view of the fact that micro-nano structures provide a new idea for the development of novel materials, the preparation of micro-nano structures and control of the geometry of micro-nano structures have ever more attracted attention, and the processing technology of micro-nano structures has been introduced. In recent years, micro-nano structures processing technology has been rapidly developed both at home and abroad, and devices prepared by micro-nano processing technology have been widely used in various fields such as national defense and the military [6,7,8], biomedicine [9,10,11], aerospace [12,13], and advanced manufacturing. At present, micro-nano machining technology has become one of the most promising research fields in the new century and has shown unlimited development potential in many disciplines.

The preparation methods of micro-nano structures can currently be roughly divided into two types. The first preparation method is to grow a new structural unit on the original structure, or build a large-size structure based on the interaction between small-size structural units. Typical preparation methods include chemical vapor deposition [14], self-assembly technology, etc. The second preparation method is to remove a certain structure from the original structure, so as to form a specific micro-nano structure unit, which mainly includes optical etching [15], plasma etching [16], an ion beam [17], ultrafast laser direct writing technology [18], and other preparation technologies. Additionally, for the capability of lasers in irradiating materials, remarkable applications of laser processes were achieved [19,20].

Chemical vapor deposition (CVD) refers to a process in which a vapor or gas reacts chemically at the gas–solid interface or in the gas phase by increasing the temperature, light radiation or other energy to produce new sediments deposited on the substrate. The CVD method has been used in the preparation of many kinds of nanostructured nanomaterials due to its advantages such as an adjustable deposition rate and controllable process parameters.

Wu et al. [21] prepared TiAlSiN surface coating on the surface of cemented carbide by the CVD deposition method, and analyzed the change in deposition pressure on the micro-nano structures of the coating surface. Zhu et al. [22] incorporated O into Ti(C,N) coatings by CO doping in the CVD deposition method, and found that the surface micro-nano structures changed from columnar and large-faceted grains to equilateral and nanoscale grains with the increase in the CO fraction.

It can be found that chemical vapor deposition can successfully prepare coatings with surface micro-nano structures, but the micro-nano structures are generally grown by self-deposition with this method, so the size and shape of the micro-nano structures are not controllable, and it is difficult to achieve the target micro-nano structures. And chemical vapor deposition will cause some degree of chemical and safety hazards.

Lithography is the most common processing method for the preparation of micro-nano patterns, which means that the pattern is transferred to the photoresist material by controlling the exposure amount, and then transferred to the base material by different kinds of etching techniques. Lithography is a high throughput technology that can achieve high resolution, so it is widely used in the preparation of micro-nano structures.

In recent years, lithography technology has broken through the limitations of traditional imprint lithography, making the preparation of lithography technology more efficient and more widely used. Zhou et al. [23] prepared a three-layer array structure with a total height of 45 microns through the triple lithography technique of a three-layer hybrid mask, so that the functional surface has the performance of reducing the drag. Kagawa et al. [24] prepared a surface micro-nano suction cup array using dip rotary UV lithography. The surface micro-nano structures inclination reached 51°, and the suction force was stronger than that of the general method. Syu et al. [25] developed a maskless lithography system, which can be patterned on the photoresist (PR) layer to prepare a large surface micro-nano structure; this makes the lithography technical steps simpler.

Although lithography technology has made many breakthroughs in recent years, current lithography technology still has some problems such as a low output, high processing cost and cumbersome operation. Each test needs to go through the steps of gluing, pre-drying, exposure, development, hardening film, etching, and degluing, etc.; the cycle is long, and each step may introduce defects for various reasons.

The ultrafast laser, due to its advantages such as high instantaneous power, high processing accuracy, and small thermal effect [26,27,28], has been widely used by researchers since its discovery, and has great development potential in various fields. Ultrafast laser direct writing is a green technology that removes the local materials by radiating the materials directly in ultrafast time, which will not produce waste gas and liquid as traditional methods do. Additionally, ultrafast laser direct writing technology easily realizes automated production. Thus, ultrafast laser direct writing technology could not only eliminate the need to buy chemicals, but also save labor. Meanwhile, ultrafast laser direct writing is suitable for industrial manufacturing due to its high precision and high efficiency. However, for enterprises, one-time investment in laser systems is considerable. This technology is more suitable for large enterprises, because mass production can quickly reach the balance of input and output, and quickly achieve profitability.

Compared to the traditional method, ultrafast laser direct writing technology, as a new processing technology, has many advantages in the preparation of micro-nano structures. Compared with traditional micro-nano structures preparation methods such as chemical vapor deposition and electrochemical preparation, ultrafast laser direct writing technology has low environmental requirements and does not need a vacuum processing environment. Ultrafast laser processing, as a non-contact processing, does not contaminate material samples and does not require chemical reagents. Its requirements for the materials to be processed are not high, and it is widely used in various dielectric materials. Compared with optical lithography, ultrafast laser processing technology is the direct processing of materials, without the need for complex process steps. Additionally, ultrafast laser direct writing technology has a simple processing operation and higher processing efficiency, which can be directly written on the surface of curved materials.

At present, many research results have been obtained for the preparation of surface micro-nano structures by laser direct writing of common materials by ultrafast laser. For example, in the study of stainless steel, Nas et al. [29], Moldova et al. [30], and Soltani-Kordshuli et al. [31] studied the effect of surface micro-nano structures on the surface wettability of stainless steel by writing points, slots, polygons and other micro-nano arrays directly by laser. The influence of surface tribology and other properties was examined. There are also many research results on the surface micro-nano structures of some semiconductor materials processed by laser. Ou et al. [32] formed a hexagonal micro-nano structure on GaN thin film by femtosecond laser ablation and realized the efficient preparation of the surface micro-nano structure of the material. Amsellem et al. [33] used a picosecond laser to process the surface micro-nano structures of SiC and studied the influence of laser parameters on the micro-nano structures.

In summary, ultrafast laser direct writing technology has made a lot of experimental progress in the preparation of surface micro-nano structures, and there are research results in various metal materials, semiconductor materials, and composite materials. Benefiting from the unique advantages of ultrafast laser processing, ultrafast laser direct writing processing technology is likely to become a new method for preparing micro-nano structure metasurfaces in the future [34,35]. However, ultrafast laser processing also has some defects and limitations. For example, due to the thermal effect of the laser, heat-affected zones will inevitably appear on the surface of the material, affecting its performance. When small nano structures are processed by ultrafast laser direct writing, the morphology is difficult to control accurately with high processing velocity. Additionally, ultrafast laser direct writing also has difficulty realizing the processing of microstructures with a high depth-to-diameter ratio. Moreover, ultrafast laser direct writing for large-format micro and nano manufacturing still faces many challenges. The processing quality consistency of an ultrafast laser direct writing surface microstructure with a large format needs to be improved.

Microwaves in the 8 to 12 GHz band are named the X-band. The X-band microwave radome is widely used in ship-borne, airborne and space-borne radar systems because of its high resolution and strong penetrating power. How to efficiently prepare X-band radomes with excellent performance and a stable structure has become a research hotspot in recent years. At present, the X-band radar radome generally uses the patch method to increase the transmittance. An array of metal patches is attached to the surface of the radome. The X-band transmittivity is increased by the resonance of the array of metal patches to the electromagnetic wave. However, the procedure of the patch method is complicated. Using the patch method is time-consuming and it is difficult to achieve the required accuracy. Although the lithography method can meet the accuracy requirements, the steps in this method are complicated and the technical requirements for operators are high. In contrast, ultrafast laser direct writing processing has become the optimal technology for achieving this goal. The required thickness of the materials layer is first plated on the surface of the base materials, and then the laser is used to etch and remove the materials layer directly. Subsequently, the required materials array is left on the surface of the base materials. This ultrafast laser direct writing method is simple to operate and has important practical significance for radar and other fields.

Today, the research into ultrafast laser direct writing technology is not mature, and there are still some defects and challenges to be solved. First of all, at this stage, most of the articles do not systematically analyze and study the specific role of the change in laser parameters on the action of materials. Secondly, there are few studies on the processing characteristics and post-processing performance analysis of laser and multilayer composite flexible thin films. In this study, for an X-band microwave radome with high resolution and strong penetrating power, taking flexible multilayer films as the research object, the picosecond laser direct writing technology, micro-nano structures design and microwave transmittance of the surface micro-nano structures of flexible wave-transparent metamaterials were studied, and the feasibility of the picosecond laser direct writing method for flexible wave-transparent metamaterials was evaluated. Firstly, the optimal laser parameters for surface machining of the composite material are obtained by adjusting different laser parameters. Then, the optimal laser parameters are used to process the micro-nano arrays of the composite films, and the influence of the surface micro-nano structures with different square column side lengths and slot widths on the X-band transmittance is analyzed.

## 2. Experimental Procedure

The material used in this study was a three-layer flexible composite film material, which was prepared by evaporating plating silicon dioxide (SiO_2_) and gold (Au) on a polyimide (PI) substrate. Firstly, a PI film with thickness of 50 µm was chosen as the substrate. Secondly, a SiO_2_ layer with thickness of 70 nm was deposited on the PI. Finally, an Au layer with thickness of 50 nm was deposited on the SiO_2_ layer. It should be noted that to ensure the binding force of the Au layer to the SiO_2_ layer, a few nanometers of nickel were deposited on the SiO_2_ layer before the depositing of the Au. The composite film (Au-SiO_2_-PI) was used for subsequent surface micro-nano structure machining.

**Figure 1:**
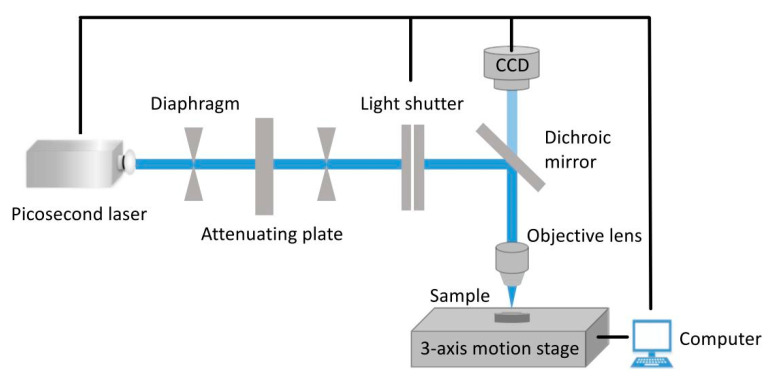
Schematic diagram of test platform.

The actual power and operating frequency of different picosecond lasers were different. The model of picosecond laser used in this paper was a GS-PIR70 infrared picosecond laser (wavelength 1064 nm, pulse width 14 ps) (Guoshen Photoelectric technology (Shanghai) Co., Ltd., Shanghai, China), and the picosecond laser processing platform built by it is shown in Figure 1. Compared with the common nanosecond laser, the picosecond laser has a smaller heat-affected zone with the material and a higher processing accuracy, so it is more suitable for the processing of micro-nano structures. Compared with other lasers, the picosecond laser has a wider adjustment range of laser parameters, which is convenient to study the effect of laser parameters on materials. In order to ensure the accuracy of laser processing, it was first necessary to explore the interaction between different laser parameters and composite materials in order to explore the best processing parameters for subsequent processing of wave-transmitting micro-nano structures. The surface of the sample was cleaned with ethanol before laser direct writing processing to eliminate surface contamination.

In the actual laser processing experiment, the main processing parameters of the laser were laser power, working frequency, scanning speed, spot diameter, pulse width, etc. The spot diameter and pulse width were limited by the laser device and therefore cannot be changed in the test. Xing et al. [36] found that if the scanning speed is too low during laser processing, the number of overlapping spots per unit area will increase, leading to severe carbonization and affecting the processing quality. When the scanning speed is too high, the precision of the laser scanning will decrease. When the scanning speed is between the two values, the laser processing effect will not differ much. In addition, the influence of the jumping speed on the laser processing result is very small. Therefore, this paper adjusts these two parameters to the appropriate parameters according to the current research results of the ultrafast laser, and does not study the influence of these two parameters on laser processing. This experiment mainly studied the laser power and laser operating frequency which have a major influence on the surface topography after processing. According to the action law of an ultrafast laser on materials and the parameter adjustment range of the picosecond laser used in this experiment, the laser parameters set in this experiment are shown in Table 1. When the laser pulse power was set to 50, 100, 150, and 200 KHz, respectively, the laser power was set to 1, 2, 3, 4, 5, 6, 7, 8, and 9 W to process flexible films, and the laser with different processing powers was processed within a circle with a radius of 1mm to explore the best processing parameters.

After processing the materials with different laser frequencies and laser power, the optimal processing parameters of the picosecond laser for the composite thin film materials were obtained, and the different micro-nano square column arrays were prepared by writing directly on the composite thin film. In this experiment, nine kinds of array structures were arranged and combined for different square column side lengths (L = 0.5 mm,0.9 mm and 1.3 mm) and different slot widths (W = 0.05 mm,0.1 mm and 0.15 mm) (L represents the side length of the square column and W represents the width of the slot), so as to specifically analyze the influence of array size changes on X-band transmittance. The size of each piece of sample is 25 mm × 12 mm.

**Figure 2:**
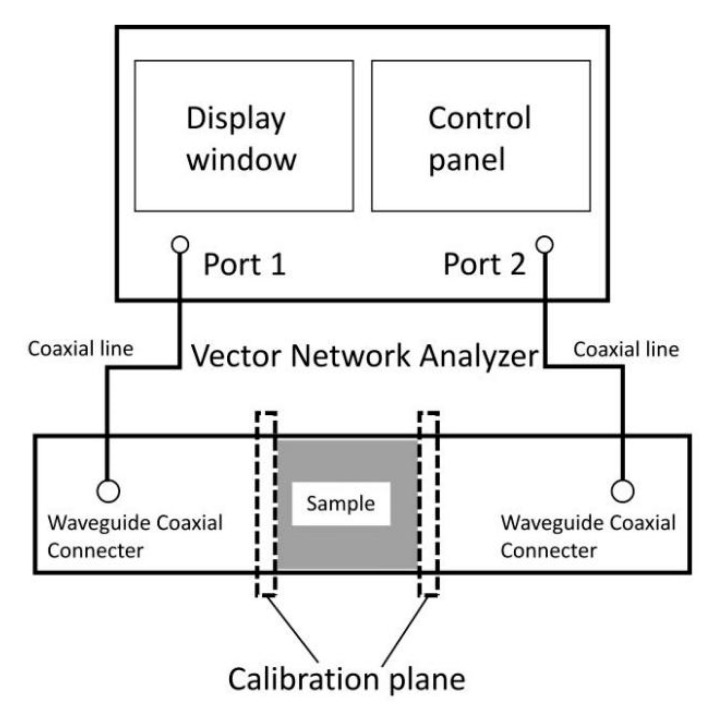
Schematic diagram of the transmittance measured by the waveguide test.

Nine groups of picosecond laser-processed samples and unprocessed samples obtained by the test were tested by the waveguide method. The schematic diagram of the waveguide method is shown in Figure 2. The equipment used in this test was a Keysight P9375A vector network analyzer (Keysight Technologies (China) Co., Ltd., Beijing, China). The test principle of the waveguide method is that the sample is placed in a waveguide measuring device, and the two ends of the waveguide device are connected to a calibrated network analyzer through a waveguide coaxial converter. The parameters of S_11_, S_22_, S_21_, and S_12_ were measured, respectively. The reflectance (R), transmittance (T), and absorptivity (A) can be calculated by the parameter S:

R = |S_11_|^2^ = |S_22_|^2^

T = |S_21_|^2^ = |S_12_|^2^

A = 1 – R − T

This study only focused on the transmittance of the material, so only two parameters (S_21_ and S_12_) need to be measured. The test band was 8.2–12.4 GHz, tested once every 0.01 GHz, and a total of 421 data were obtained for each set of data, and each sample was tested twice.

In addition, the machined surface micro-nano structures were observed and analyzed under an OLYMPUS BX53M optical microscope (Olympus (China) Co., Ltd., Beijing, China). Observation of the micro-nano structures was conducted using COXEM EM-30AX model scanning electron microscopy (SEM) equipped with an energy dispersive spectrometer (EDS) (Coxem kosdaq listed company, Daejeon, Republic of Korea). The micro-nano grooves were observed with a Keyence VK-X3000 shape measurement laser microscopic system (Keyence (China) Co., Ltd., Shanghai, China).

## 3. Results and Discussion

### 3.1. Film Thickness and Elements

**Figure 3:**
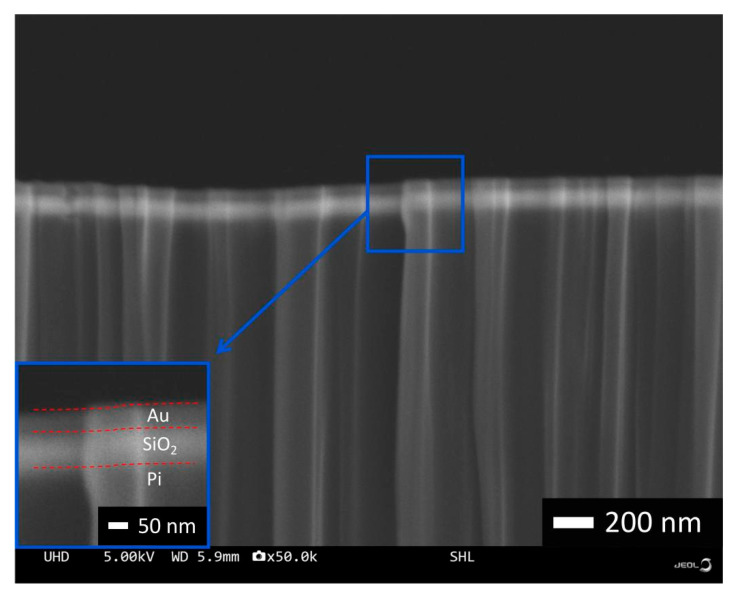
Observation of cross-section of flexible film by SEM.

The cross-section micro-nano structures of the flexible composite film are shown in Figure 3. Under the SEM image, it can be clearly seen that the composite film is divided into three layers, and the thickness of the polyimide substrate at the bottom layer is 50 µm. As the substrate of the composite flexible film, it is the key for the composite material to become a flexible material. In addition, the high stability of the polyimide itself is also a factor for choosing the material as the substrate. The middle silica layer has a thickness of 72 nm. As the middle layer, the silica layer is used to separate the upper and lower layers and is the key layer for the subsequent transmittance structure. The thickness of the top layer of gold film is 50 nm, and the laser-processing surface micro-nano structures are used mainly to process the surface of the gold layer.

**Figure 4:**
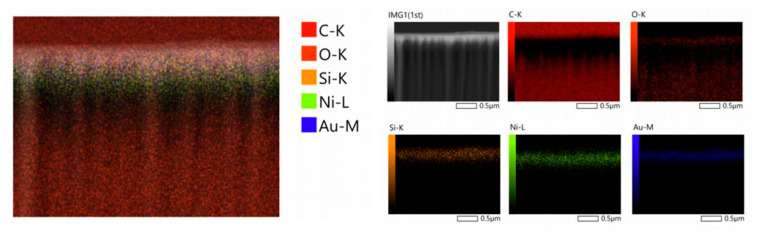
EDS energy dispersion spectra of flexible films.

The EDS energy dispersion spectra clearly show the general distribution of different elements in the cross-section of the film. As can be seen from Figure 4, in addition to the gold element in the surface layer and the silicon element in the middle layer, the nickel element was also found from the analysis of the detection, which was doped in the middle of the gold layer and the SiO_2_ layer. This is because the composite film is prepared by vapor deposition. If the film is directly gilded onto the SiO_2_ layer, the gold adhesion is very poor and it easily falls off. Therefore, a layer of Ni of about a few nanometers is first plated on the surface when preparing, and then gold-plated, so that the gold layer on the surface can be firmly attached to the material.

### 3.2. Influence of Laser Parameters on Material Surface Morphology

Laser parameters determine the machining quality of the composite surface. The micro-nano structures of the material surface are greatly affected by different laser parameters. Only when the appropriate laser parameters are determined can the follow-up test be carried out.

**Figure 5:**
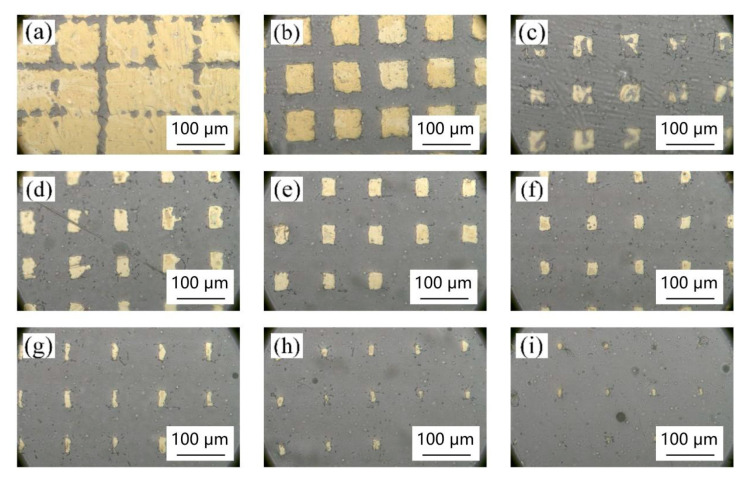
Surface morphology of different powers at 50 KHz operating frequency: (**a**) 1 W, (**b**) 2 W, (**c**) 3 W, (**d**) 4 W, (**e**) 5 W, (**f**) 6 W, (**g**) 7 W, (**h**) 8 W, and (**i**) 9 W.

Figure 5 shows the surface morphology of the composite thin film material at the picosecond laser operating frequency of 50 KHz. When the laser power increases from 1 W to 9 W, the slot width gradually increases. Correspondingly, the gold ablation of the surface of the composite thin film by the laser becomes more and more serious, and the heat-affected area becomes larger and larger. At 9 W power, there is almost no gold on the surface of the composite. At 1 W power, the laser energy is too small, and only shallow marks can be etched on the surface of the composite film, and it is discontinuous. When the laser power is 2 W at the operating frequency of 50 KHz, the structure of the prepared micro square column is relatively complete, and there is no excessive ablation.

**Figure 6:**
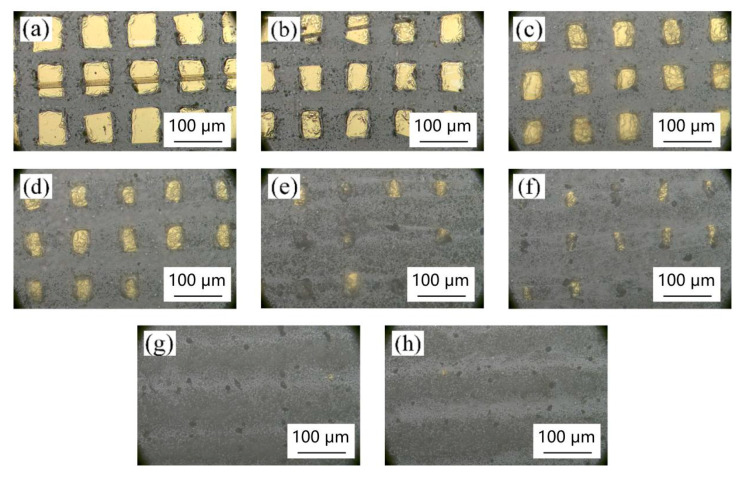
Surface morphology of different powers at 100 KHz: (**a**) 2 W, (**b**) 3 W, (**c**) 4 W, (**d**) 5 W, (**e**) 6 W, (**f**) 7 W, (**g**) 8 W, and (**h**) 9 W.

**Figure 7:**
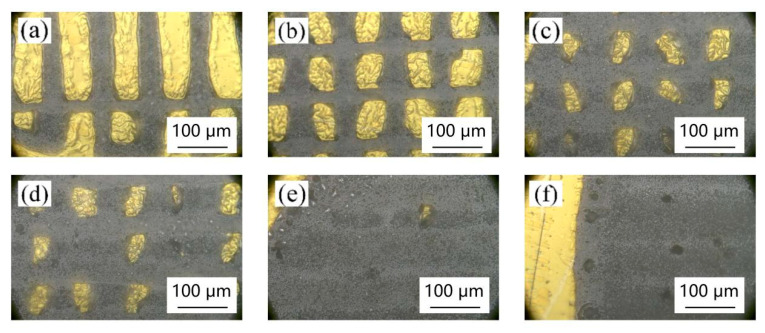
Surface morphology of different powers at 150 KHz operating frequency: (**a**) 4 W, (**b**) 5 W, (**c**) 6 W, (**d**) 7 W, (**e**) 8 W, and (**f**) 9 W.

**Figure 8:**
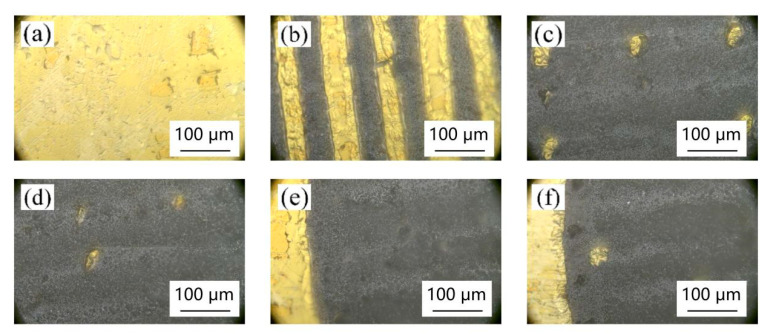
Surface morphology of different powers at 200 KHz operating frequency: (**a**) 4 W, (**b**) 5 W, (**c**) 6 W, (**d**) 7 W, (**e**) 8 W, and (**f**) 9 W.

Figure 5, Figure 6, Figure 7 and Figure 8 show the effect of different laser parameters on the composite material. It can be found that under the same operating frequency, the higher the laser power is, the greater is the ablation of the composite material and the larger is the heat-affected zone. This is consistent with the theoretical analysis. With the same frequency, the greater the laser power is, the higher is the laser energy on the materials. The materials on the surface radiated by the laser will be vaporized, melted, turned into plasma, etc., and finally separated by the laser ablation. The greater the laser energy is, the larger is the area of ablation. Regardless of the laser operating frequency, when the laser power reaches up to 9 W, the gold layer on the surface of the composite film was almost all ablated. The higher the laser operating frequency is, the smaller is the ablation of the composite film at the same low power. At 150 KHz and 200 KHz, when the laser power reaches up to 4 W, the laser begins to produce ablation marks on the surface of the composite film. When the laser power is lower than 3 W, the processing marks on the surface of the composite film are not observed.

Figure 8 shows the surface morphology of the composite thin film material at the picosecond laser operating frequency of 200 KHz. It can be seen from the figure that no matter how the laser power is changed at the operating frequency of 200 KHz, a relatively regular micro-nano structure cannot be prepared. When the power rises to 4 W, slight etching marks begin to appear on the surface of the material, as shown in Figure 8a. From Figure 8b, it can be seen that under the power of 5 W, the laser direct writing only has longitudinal etching micro-grooves, while the transverse etching marks are only slight, and the surface gold layer is not etched completely, indicating that the power is still too small. When the laser power reaches 6 W, as shown in Figure 8c, the surface of the composite film is too much etched by laser, and the gold layer on the surface is almost completely etched; this cannot meet the design requirements, indicating that the power is too great. Therefore, better surface micro-nano structures cannot be prepared with this picosecond laser at a 200 KHz operating frequency.

By comparing and analyzing the micro-morphology under all working parameters, it can be seen that Figure 5b and Figure 6b are the two samples with an ideal microscopic morphology. After further comparison, it is found that when the laser power is set to 100 KHz and 2 W, the metasurface micro-nano structures obtained by laser treatment are more complete, and the prepared square column and slot width are more in line with the test expectation.

**Figure 9:**
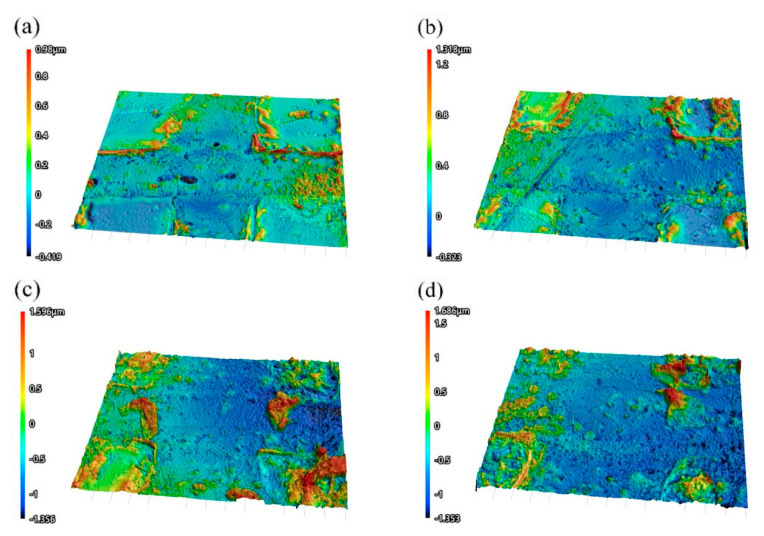
The three-dimensional morphology of micro-nano grooves under different powers at 100 KHz operating frequency: (**a**) 2 W, (**b**) 3 W, (**c**) 4 W, and (**d**) 5 W.

Figure 9 shows the three-dimensional dimensions of the microslots on the surface of the material after direct writing of different laser parameters under the confocal laser microscope. It can be seen that when the laser power rises from 2 W to 5 W, the width and depth of the micro-groove gradually increase, in line with the law of laser processing. The gold layer thickness of the most surface layer of the multilayer composite is 50 nm, and the groove depth of the laser machining micro-nano groove is at least 277 nm. Therefore, as long as a clear and uniform groove can be etched, the groove depth can meet the requirements.

### 3.3. Microstructure Analysis of Samples with Different Micro-Nano Structures

**Figure 10:**
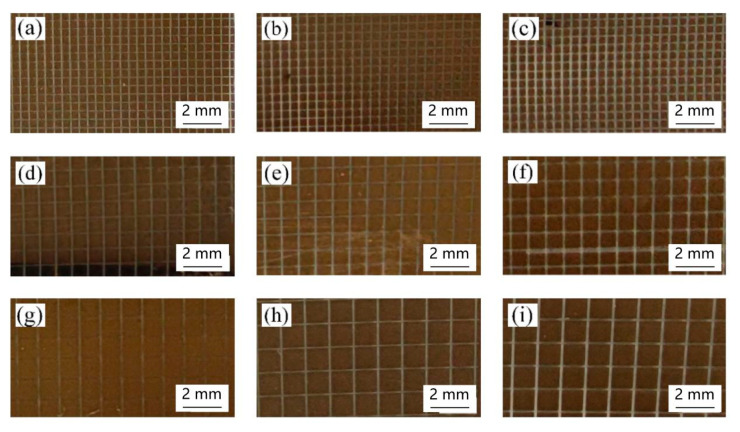
Macroscopic picture of sample by picosecond laser processing: (**a**) L = 0.5 mm, W = 0.05 mm, (**b**) L = 0.5 mm, W = 0.1 mm, (**c**) L = 0.5 mm, W = 0.15 mm, (**d**) L = 0.9 mm, W = 0.05 mm, (**e**) L = 0.9 mm, W = 0.1 mm, (**f**) L = 0.9 mm, W = 0.15 mm, (**g**) L = 1.3 mm, W = 0.05 mm, (**h**) L = 1.3 mm, W = 0.1 mm, and (**i**) L = 1.3 mm, W = 0.15 mm.

Nine micro-nano structures samples with a side length (L) of the square column from 0.5 to 1.3 mm and with a groove width (W) of the square column from 0.05 to 0.15 mm by picosecond laser processing are presented in Figure 10. It can be seen that square column arrays with a regular shape can be obtained by using ultrafast laser direct writing technology.

**Figure 11:**
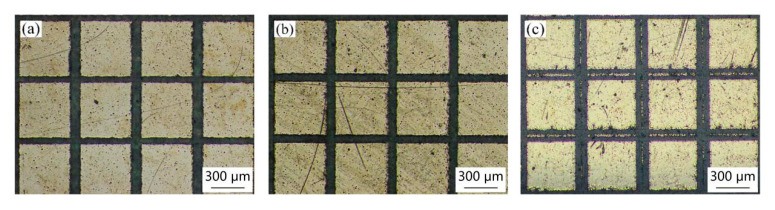
Surface morphology of different groove widths when the side length of the square column is 0.5 mm: (**a**) 0.05 mm, (**b**) 0.1 mm, and (**c**) 0.15 mm.

**Figure 12:**
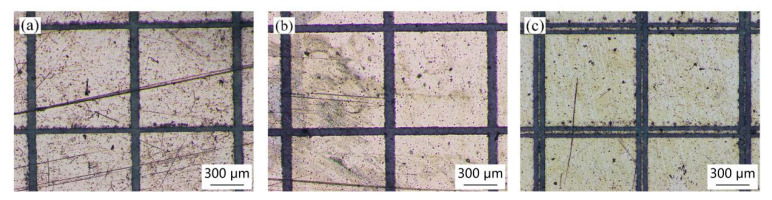
Surface morphology of different groove widths when the side length of the square column is 0.9 mm: (**a**) 0.05 mm, (**b**) 0.1 mm, and (**c**) 0.15 mm.

**Figure 13:**
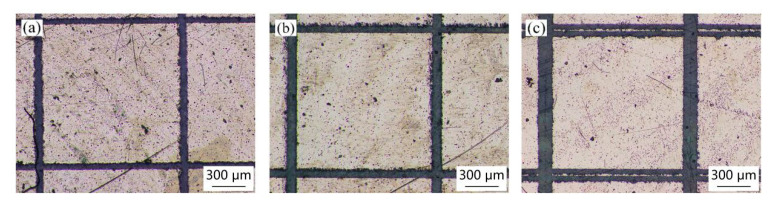
Surface morphology of different groove widths when the side length of the square column is 1.3 mm: (**a**) 0.05 mm, (**b**) 0.1 mm, and (**c**) 0.15 mm.

Figure 11, Figure 12 and Figure 13 present the micromorphology of the array structures machined by picosecond laser direct writing. It can be seen that the square column shape is regular and the micro structures meet the design requirements. The square column sides are straight without obvious defects.

**Figure 14:**
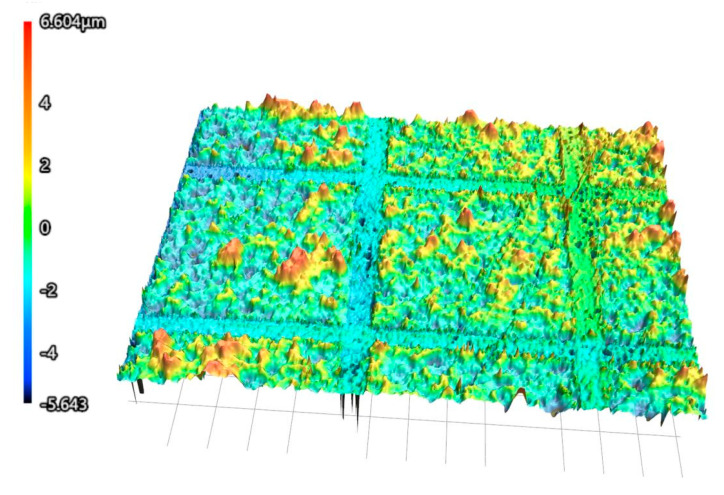
Three-dimensional topography of surface micro-nano structures.

Through the observation analysis of the shape measurement laser microscopy system, we can see from Figure 14 that the height of the square column is 12.247 microns. This shows that the top layer of the gold layer and the middle layer of the silica layer are etched by laser, and the consistency of the groove morphology is also good, which meets the processing requirements.

### 3.4. Analysis of Wave Transmittance

**Figure 15:**
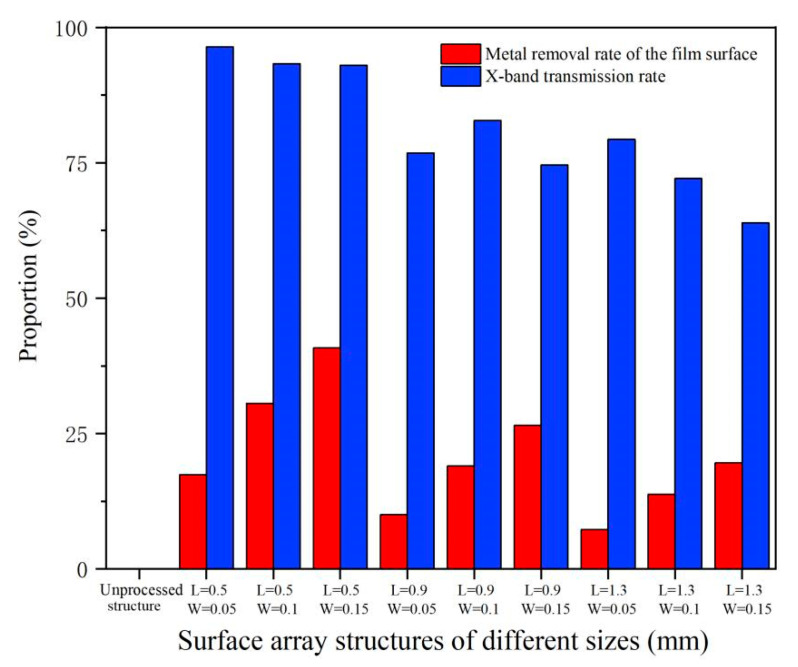
Surface metal removal rate of different micro-nano arrays and corresponding X-band transmittance.

The X-band transmittance of flexible composite materials is a direct method to evaluate the feasibility of the picosecond laser direct writing method for flexible wave transmittance surface micro-nano structures. By exploring the transmittance of micro-nano structures of the nine different array combinations, the optimal array parameters can be selected. In order to explore the overall law, the arithmetic average value of each group of transmittance data is first analyzed, and the obtained data are shown in Figure 15. The figure calculated the metal removal of the surface of the composite material by laser when different micro-nano array structures were prepared. The materials removal rate of the surface of the film and the X-band transmittance of different micro and nano arrays obtained from the test were compared and analyzed in a graph.

Through the analysis of the table content data, it can be seen that when the surface of the film sample is not laser-processed, the metal removal rate is zero, and the wave transmittibility in the 8–12 GHz band is only 0.007%, which can be identified as the unprocessed composite film that is not wave-transmittable for the X-band.

According to the analysis in Figure 14, we can see that the metal removal rate has no obvious law for the slot width transmittance, and it is not the case that the higher the metal removal rate of the film surface is, the better is the X-band transmittance. The morphology characteristics of the micro-nano structures are the main factors affecting the film transmittance. The penetration structures with a side length of 0.5 mm and a groove width of 0.05 mm and a side length of 1.3 mm and groove width of 0.15 mm are both about 20% of the metal removal rate, but the transmittance of the side length of 0.5 mm and groove width of 0.05 mm is as high as 96.4%; these are the best data of this picosecond test. The micro-nano structures with a side length of 1.3 mm and a slot width of 0.15 mm were the worst at 63.9%.

**Figure 16:**
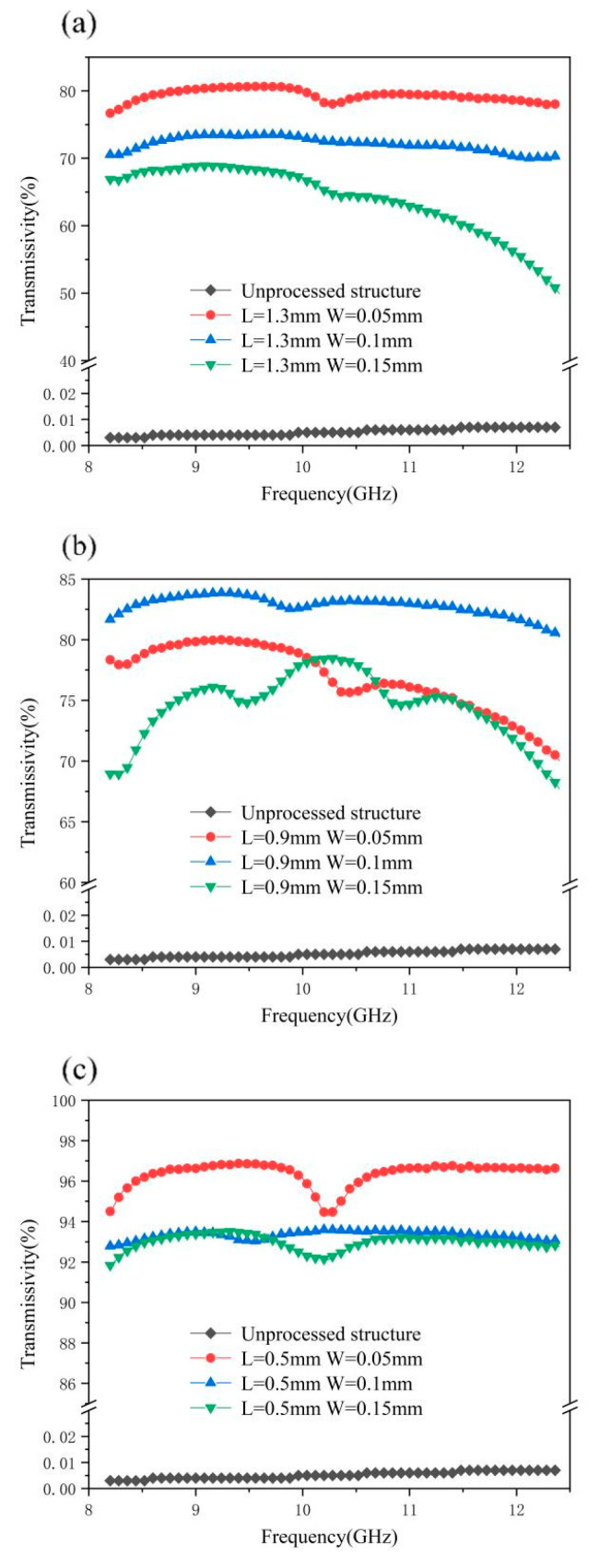
Transmittance chart of micro and nano structures with different slot widths under the same square column side length: (**a**) transmittance chart of metasurface square column side length 1.3 mm, (**b**) transmittance chart of metasurface square column side length 0.9 mm, and (**c**) transmittance length of metasurface 0.5 mm.

From Figure 16, we can clearly see the overall curve distribution and trend. It can be clearly seen from the figure that after the laser processing of the surface micro-nano structures, the X-band transmittance of the material has been significantly improved, and the worst transmittance has reached about 50%, while the other samples have a transmittance of more than 70%, which indicates that the designed micro-nano structures array is very effective at improving the transmittance of the X-band. As can be seen from Figure 16a, when the side length of the square column is set to 1.3 mm, the wave transmittance does not reach 90%, and the fluctuation range of the three curves in Figure 16b is large, ranging from 65% to 85%. It can be seen from Figure 16c that when the square column side length is 0.5 mm, the X-band transmittance exceeds 90%, and the distribution curve is relatively stable with a small fluctuation range. As can be seen from Figure 16, the side length of the square column is the main factor determining the quality of the wave transmittance, and the slot width also has an impact on the wave transmittance, but the impact is not as great as the side length of the square column.

In the case of the same square column side length setting, the smaller the slot width setting is, the greater is the transmittance at 8–12 GHZ. When the parameters are set as a 0.5 mm square column side length and a 0.05 mm slot width, the wave transmission efficiency is as high as 96.4%; these are the best data of the wave transmission efficiency of this picosecond test.

**Figure 17:**
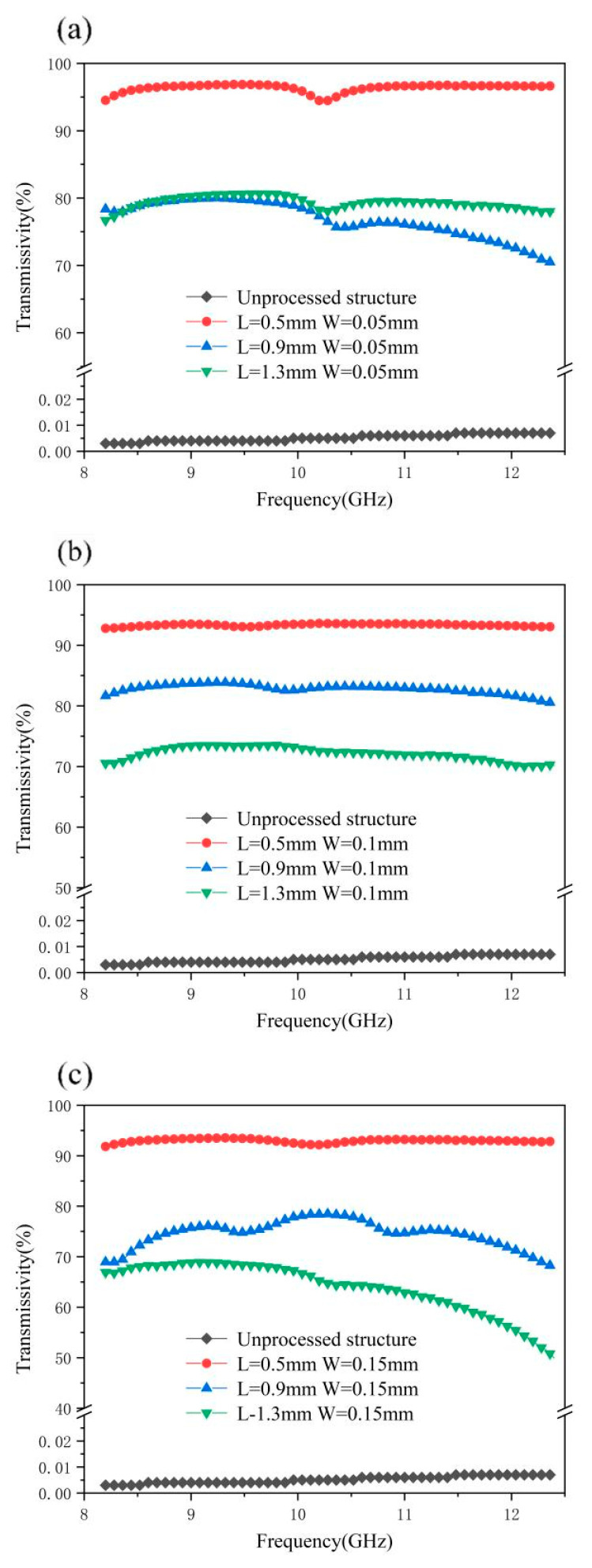
Transmittance chart of micro and nano structures with different square column side lengths under the same slot width: (**a**) transmittance diagram of metasurface groove width of 0.05 mm, (**b**) transmittance diagram of metasurface groove width of 0.1 mm, and (**c**) transmittance length of metasurface square column of 0.15 mm.

Figure 17 shows the wave transmittance of different square column side lengths with a fixed slot width. It can be seen from the figure that, under a fixed slot width, the wave transmittance of different square column side lengths is successively distributed in different areas, and the wave transmittance is 1.3 mm, 0.9 mm, and 0.5 mm from low to high. However, 0.9 mm is shown in Figure 17a. The curve of the transmittance of 1.3 mm is slightly different, and the two curves have a similar distribution and cross first. The curve distribution in Figure 17b is the most intuitive. The three curves are about 72%, 82%, and 93%, respectively, and the curve fluctuation is not large. This further shows that the length of the square column is the main factor affecting the transmittance of the X-band compared with the width of the slot.

In addition, from Figure 16 and Figure 17, we can see that the transmittance curve is basically concave between 10 and 11GHz, which is the central frequency of the transmission passband. In order to meet the requirements of the X-band transmission, the transmission passband of the designed surface array structure contains 8–12GHz, and the metal patch structure is capacitive and has low-pass filtering characteristics, so there will be dents in the frequency band.

## 4. Conclusions

The objective of this paper is to investigate the feasibility of a picosecond laser direct writing process to fabricate micro-nano structures on the surface of flexible thin film in order to improve the X-band transmittance. Based on the research results and discussion, the following conclusions were obtained:

(1) In this paper, to solve the problem of traditional artificial microstructure preparation with mask, low-efficiency, long-process, high-efficiency, and high-precision laser direct writing technology was developed to prepare the X-band transmittance artificial microstructure with three-layer flexible composite film materials (Au-SiO_2_-PI). The X-band transmittance of the flexible composite film materials reached up to 90% by using the laser direct writing technology. The relevant research results provide a theoretical basis and data support for the efficient manufacturing of radome.

(2) Excellent microstructure morphology of the square columnar arrays without burning through or destroying were obtained by laser direct writing with a pulse power and frequency of 2 W and 100 KHz, respectively.

(3) When the pulse frequency of the laser was equal to or larger than 150 KHz, the square columnar micro-nano structure could not be acquired due to the over-ablation of the Au by the picosecond laser. However, when the pulse frequency of the laser was 50 KHz, the square columnar micro-nano structure could not be obtained with a laser power of 1 W or higher than 3 W because of insufficient or excessive laser energy.

(4) The X-band transmittance was effected by the characteristic of the square columnar array on the surface of the flexible thin film. The X-band transmittance was significantly increased with decreasing the length of the square column on the surface of the flexible thin film. The X-band transmittance reached up to 90% when the length of the square column on the surface of the flexible thin film was 0.5 mm. The X-band transmittance slightly increased when we decreased the width of the groove of the square column on the surface of the flexible thin film. In addition, the X-band transmittance is not proportional to the metal removal rate of the film surface.

In the future, some research work could be carried out to compensate for the limitations of this paper. Firstly, a finite–difference time–domain method could be used to simulate the microstructure and its transmittance of the flexible thin film. Secondly, femtosecond laser direct writing of the flexible thin film could be investigated. Finally, ultrafast laser direct writing of multilayer metal and dielectric films could be analyzed.

## Figures and Tables

**Table 1:** Laser setting performance parameters.

Parameter	Unit	Value
Spot diameter	μm	50
Pulse width	ps	14
Scanning speed	mm/s	1000
Jump speed	mm/s	1000
Laser power	W	1–9
Laser frequency	KHz	50–200

## Data Availability

The original contributions presented in this study are included in the article. Further inquiries can be directed to the corresponding author.
